# Epithelial–myoepithelial carcinoma of the parotid gland with primary lung cancer: A rare case report

**DOI:** 10.1097/MD.0000000000022483

**Published:** 2020-10-02

**Authors:** Huanhuan Wang, Yuyu Zhang, Bin Wang, Jinlong Wei, Rui Ji, Lihua Dong, Xin Jiang

**Affiliations:** a Department of Radiation Oncology; b Jilin Provincial Key Laboratory of Radiation Oncology and Therapy, The First Hospital of Jilin University; c NHC Key Laboratory of Radiobiology, School of Public Health, Jilin University, Changchun, China; d Department of Biology, Valencia college, Orlando, FL 32825, USA.

**Keywords:** Epithelial–myoepithelial carcinoma, salivary gland tumors, lung, adenocarcinoma, secondary primary malignancies

## Abstract

**Introduction:**

Epithelial–myoepithelial carcinoma (EMC) is a rare, low-grade malignancy that occurs primarily in the parotid gland and is most common in women aged 60 to 70 years. Cases of parotid EMC have been reported previously. Furthermore, some studies have suggested an increased risk of salivary gland tumors with secondary primary malignancies. There have been few reports of parotid EMC with other primary tumors.

**Patient concerns:**

A 62-year-old Chinese man visited the hospital with a complaint of a mass on his left cheek that had persisted for 20 years. Routine pulmonary computed tomography showed a local ground glass shadow in the lower lobe of the right lung.

**Diagnosis:**

The pathological diagnosis of lung was right lower lobe lung adenocarcinoma (pT1N0). Immunohistochemistry analysis showed that cytokeratin (CK)-7, NapsinA, and thyroid transcription factor-1 tested positive, while CK5/6, P40, and ALKD5F3 tested negative. The pathological diagnosis of left parotid gland: EMC. On immunohistochemistry staining, the outer cells expressed myoepithelial markers, such as CK5/6, P63, smooth muscle actin, while the inner cells expressed glandular epithelial markers, such as low-molecular-weight CK7 and CK8.

**Interventions:**

The patient underwent resection of the lung and parotid tumors, and received preventive radiotherapy in the parotid gland area.

**Outcomes:**

The patient is in good condition. No symptom recurrence, distant metastatic spread or significant toxicity occurred during or after the treatment. The patient remains under regular surveillance.

**Conclusion:**

We report a rare case of parotid EMC with a second primary lung adenocarcinoma. This case is the third case of primary lung cancer associated with parotid EMC reported to date and the first to be reported in nearly 30 years.

## 1 Introduction

Epithelial–myoepithelial carcinoma (EMC) is a rare, low-grade malignancy, accounting for about 0.5% of all salivary gland malignancies. EMC mainly occurs in the parotid gland, while only a few cases occur in the submandibular gland and other small salivary glands. EMC mainly occurs in women aged 60 to 70 years. Histologically, EMC has a typical 2-layer histological structure. The inner layer is composed of epithelial cells, while the outer layer is composed of myoepithelial cells, which are glandular and arranged in a duct-like pattern. There are many reports of single cases of parotid EMC,[^1^,^2^,^3^] but there are few reports of simultaneous dual primary parotid EMC and other tumors. Here, we report a rare case of parotid EMC with a second primary lung adenocarcinoma. A previous study of 349 women with salivary gland tumors found that the incidence of breast cancer was twice as high as expected.^[4]^ Another study indicated that 825 patients with primary salivary gland tumors over a 15-year period exhibited a significant increase in the incidence of secondary primary tumors. Among them, the tumors mainly occurred in the breast in female patients and in the prostate in male patients. Therefore, the study points to an association between salivary gland tumors and other hormone-dependent tumors.^[5]^ Cases of other parotid gland tumors with secondary primary tumors have been reported previously, but only 2 cases of parotid gland EMC with other secondary primary tumors have been reported.[^6^,^7^] The history, imaging findings, histopathology results, and subsequent treatment of a patient are reported in this paper. The purpose of this paper is to further understand EMC and associated secondary primary tumors.

## 2 Case presentation

In December 2018, a 62-year-old Chinese man visited the hospital with a complaint of a mass on his left cheek that had persisted for 20 years. The mass was initially the size of a pea and asymptomatic. In the most recent 7 years, the tumor gradually increased to about the size of an egg, without tenderness and occasionally with acid swelling. He had no previous history of surgery and had a history of hepatitis B, heavy smoking, and alcohol consumption. Physical examination showed that the tumor of the left parotid gland was tough, about 4.0 × 3.0 cm in size, with good mobility, no tenderness, clear boundaries, and no symptoms of facial paralysis.

**Figure 1:**
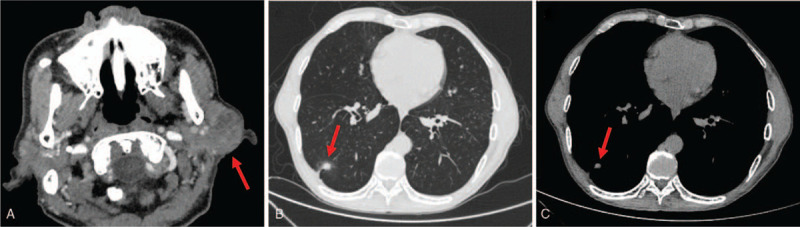
A: The left parotid gland area presented a lumpy mixed density shadow, with a size of 3.1 × 2.5 cm, unclear boundary, and low density of the internal center of the mass; B, C: 2.3 × 2.0 cm shadow in the lower lobe of the right lung, with local traction adjacent to the pleura.

Ultrasonography of the parotid gland suggested a mixed mass of the left parotid gland, with a size of about 3.29 × 2.48 cm, unclear boundaries, regular shape, slightly lower echo inside the parenchyma, uneven distribution, a large number of thick and small calcified plaques, and sparse blood flow signal. Ultrasonography thus indicated the presence of a mixed parotid gland tumor. Contrast-enhanced computed tomography (CT) of the parotid gland showed a lumpy, mixed-density shadow in the left parotid gland area, with a size of 3.1 × 2.5 cm, uneven enhancement with gadolinium contrast, and unclear boundaries. In addition, the density of the internal center of the mass was low, and a small calcification shadow was observed (Fig. 1A). Therefore, the patient wished to remove the parotid gland mass by surgery. However, preoperative routine pulmonary CT showed a local ground glass shadow in the lower lobe of the right lung, about 2.3 × 2.0 cm in size, with small solid nodules and empty bubbles, and local traction of the adjacent pleura. CT indicated the presence of a malignant lesion (Fig. 1B, C). Serum tumor markers were detected within the normal range. Other serological examinations showed no abnormalities. Head magnetic resonance imaging, abdominal/pelvic CT, and systemic bone imaging also showed no abnormalities.

**Figure 2:**
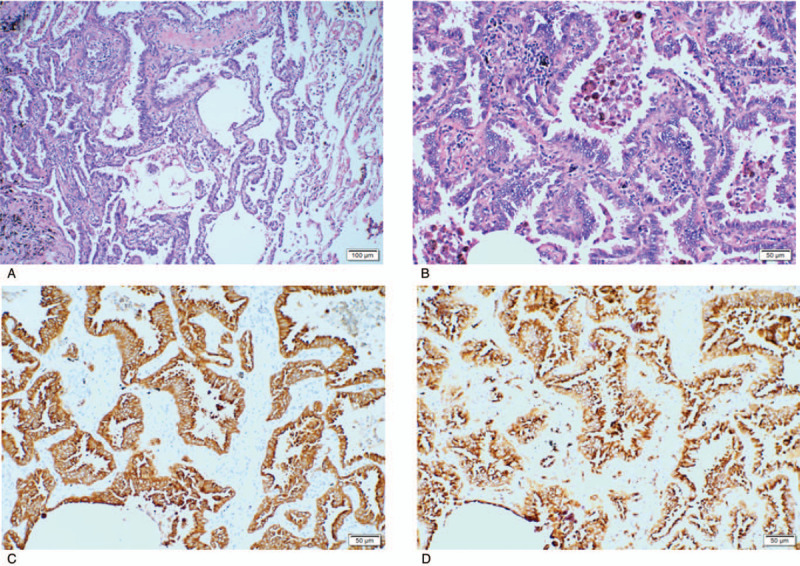
A, B: H&E, the tumor mainly consist of lepidic adenocarcinoma (about 90%), this variant typically consist of bland pneumocytic cells (type II pneumocytics or clara cells) growing along surface of the alveolar walls; C, D: IHC, the component of invasive adenocarcinoma in this case was about 10%. CK7 and NAPSINA were negative shown in figure C, D respectively. CK = cytokeratin.

As lung CT found more important lesions than the parotid mass, priority was given to the right lower lobe tumor for radical surgery under a thoracoscope. During the surgery, a 2.0 × 2.0-cm mass was touched and removed from the basal segment of the lower lobe of the right lung. The postoperative pathological diagnosis was invasive adenocarcinoma in the right lung, with adherent type 90% and acinar type 10%. There was no invasion of cancer tissue in the vascular and nerve tissue, visceral pleura, bronchial wall, bronchial resection margin, and vascular resection margin. No cancer tissues were found in the para-bronchial lymph nodes (0/7). Microscopically, the tumor mainly consisted of lepidic adenocarcinoma (about 90%); this variant typically consists of bland pneumocytic cells (type II pneumocytes or clara cells) growing along the surface of the alveolar walls, similar to the morphology defined in the sections of minimally invasive adenocarcinoma and adenocarcinoma in situ. Immunohistochemistry analysis showed that cytokeratin (CK)-7, NapsinA, and thyroid transcription factor-1 tested positive, while CK5/6, P40, and ALKD5F3 tested negative (Fig. 2A-D).

**Figure 3:**
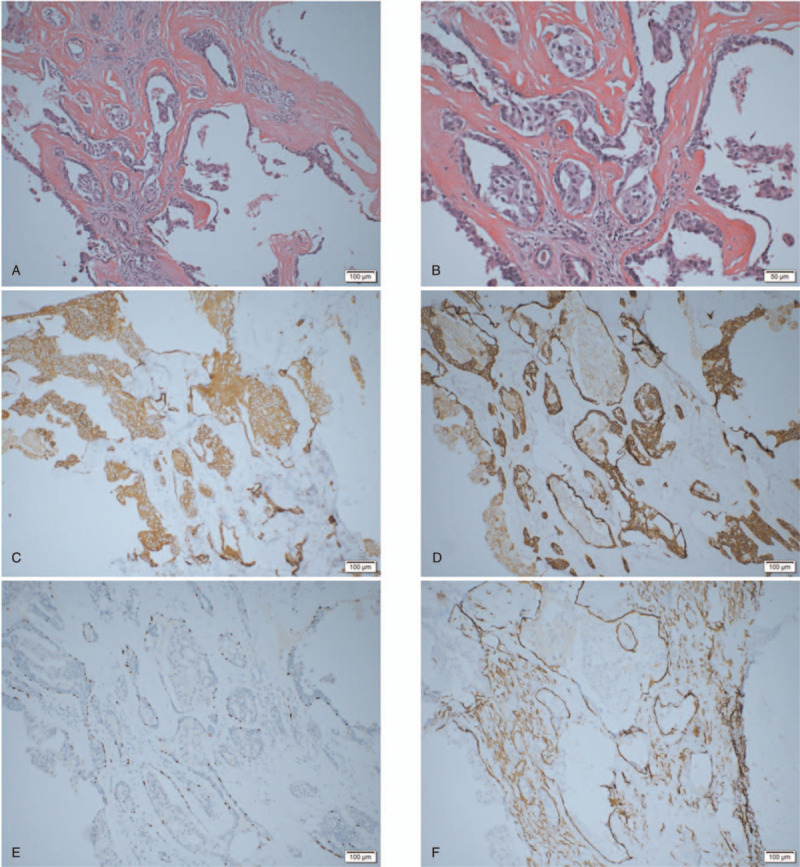
A, B: H&E, the glandular cavity or solid nests consisted of 2 layers: the inner comprised small cuboidal or low columnar cells with generally eosinophilic cytoplasm, and was surrounded by an outer mantle of cells, almost all of which had clear cytoplasm; C: IHC, The inner cells expressed glandular epithelial marker, such as low molecular weight keratin CK7, the outer cells expressed myoepithelial markers, such as CK5/6 (D), P63 (E), SMA (F). CK = cytokeratin, SMA = smooth muscle actin.

Therefore, the pathological stage was pT1N0 based on the 8th American Joint Committee on Cancer staging system. Because of the early stage of right lower lobe lung adenocarcinoma, no other adjuvant therapy was needed after surgery. He was then readmitted for the left parotid gland mass resection. During the surgery, a 4.0 × 3.0-cm tough mass was found in the superficial lobe of the parotid gland, with obvious adhesion to the surrounding tissues. The completely resected tumor was submitted for pathological examination. The neoplasms were nodular, mostly enveloped, pale in section, firm, and tough. The tumor had a localized lumen with a tan, slightly viscous fluid. Postoperative pathology assessment showed a microinvasive carcinoma of the left parotid gland: EMC. The tumor was a pleomorphic adenoma with partial epithelial cancer. The tumor volume was 3 × 2.5 × 2.2 cm. The carcinoma invaded the surrounding soft tissue and parotid gland, and the focal cancer tissue was close to the incisional margin. The nerve was infiltrated by the carcinoma, and the vasculature was negative. Macroscopically, the tumor was well circumscribed and massed with a grey cut surface. The nuclei in both cell types were single with evenly dispersed chromatin and rare, almost inconspicuous nucleoli. On immunohistochemistry staining, the outer cells expressed myoepithelial markers, such as CK5/6, P63, smooth muscle actin, while the inner cells expressed glandular epithelial markers, such as low-molecular-weight CK7 and CK8, among others (Fig. 3A-F).

Because the tumor tissue was near the incisional edge, and the nerve was infiltrated by the tumor, the patient received preventive radiotherapy in the parotid gland area 1 month after the surgery. The rapid arc technique was used for radiotherapy. Under a Varian linear accelerator, 6-MV X-ray external irradiation was applied to the area of the tumor bed. The total dose was 6000 cGy, with 200 cGy each time, administered 5 times a week. During radiotherapy, only a degree I buccal mucosa reaction occurred, without obvious dry mouth and other adverse reactions. After radiotherapy, the patient's lungs and parotid gland area were well controlled. No local progress or new lesions were found 6 months after completing radiotherapy.

## 3 Discussion

Previously, EMC was considered to be a benign tumor derived from duct cells, known as cellular adenoma.^[8]^ With the gradual deepening of our understanding of EMC, at present, EMC is considered to be a malignant tumor with a low degree of malignancy and biphasic morphology. EMC was named in 1972 by Donath et al^[9]^, and the World Health Organization classified it as a low-level salivary gland malignancy in 1991. EMC accounts for 0.5–1% of all salivary gland tumors,^[10]^ mainly occurring in the parotid gland. Due to the high degree of differentiation and low degree of malignancy of EMC cells, lymph node metastasis^[11]^ and distant metastasis are rare, but local recurrence occurs frequently.[^12^,^13^]

Histological and immunohistochemical analyses are commonly used to determine the diagnosis of EMC. Histologically, the characteristic double-layer structure is composed of inner vascular endothelial cells and outer myoepithelial cells, representing a tubule-like structure.[^14^,^15^] The diagnosis should be distinguished from cystic carcinoma, mixed adenoma, and myoepithelial carcinoma.^[16]^ On immunohistochemical staining, the inner epithelial cells show positivity for CK-7, and the outer myoepithelium shows positivity for smooth muscle actin and P63, among others. Therefore, in this case, based on the combined microscopic phenotype and immunohistochemical results, the patient was diagnosed with parotid gland EMC.

Due to the high degree of EMC cell differentiation, the tumor exhibits low-grade malignancy. Therefore, if complete resection is performed and a sufficiently safe distance is present, there is no need for subsequent adjuvant therapy. However, due to the limitation of the anatomical location of the tumors, an adequate safe distance is often not guaranteed. Therefore, postoperative adjuvant therapy is very important. Previous studies have shown that postoperative radiotherapy is highly beneficial in enhancing local control of the tumor.[^1^,^17^] However, the role of chemotherapy in adjuvant therapy is unclear.[^10^,^18^,^19^] In this case, the tumor was close to the incisional margin and invaded the nerve. Therefore, postoperative adjuvant radiotherapy was very necessary.

Previous studies have suggested an increased risk of secondary primary tumors in patients with salivary adenocarcinoma, but no firm conclusions have been drawn. In 1969, a retrospective study of 297 patients with salivary adenocarcinoma found no association between salivary adenocarcinoma and secondary primary tumors.^[20]^ However, a report of a large 15-year sample study of 825 patients was subsequently published.^[5]^ The authors of the latter arrived at the opposite conclusion; their study found a large number of secondary primary tumors in women who were more likely to develop breast cancer. Therefore, salivary gland tumors were possibility associated with a higher risk of secondary primary tumors. Parotid EMC itself is a rare tumor, and there are few reports of secondary primary tumors. Therefore, we reviewed parotid EMC and secondary primary tumors in the most recent 30 years in the hope of promoting further understanding of these tumors.

## Acknowledgments

We would like to thank Editage (www.editage.cn) for English language editing.

## Author contributions

Conceptualization, X. J. and LH. D.; Software, B. W.; Validation, X. J. and LH. D.; Formal Analysis, B. W.; Investigation, HH. W.; Resources, B. W.; Writing-Original Draft Preparation, B. W., Q, Z.and C. Q.; Writing-Review & Editing, R.J. and X. J.; Funding Acquisition, X. J.. All authors read and approved the manuscript.
